# MotomiRs: miRNAs in Motor Neuron Function and Disease

**DOI:** 10.3389/fnmol.2017.00127

**Published:** 2017-05-04

**Authors:** Zachary C. E. Hawley, Danae Campos-Melo, Cristian A. Droppelmann, Michael J. Strong

**Affiliations:** ^1^Molecular Medicine Group, Robarts Research Institute, Schulich School of Medicine and Dentistry, Western UniversityLondon, ON, Canada; ^2^Department of Pathology, Schulich School of Medicine and Dentistry, Western UniversityLondon, ON, Canada; ^3^Department of Clinical Neurological Sciences, Schulich School of Medicine and Dentistry, Western UniversityLondon, ON, Canada

**Keywords:** motor neurons, miRNA profiling, RNA stability, neurodegeneration, motor neuron disease, amyotrophic lateral sclerosis (ALS), spinal muscular atrophy (SMA), neurofilament proteins

## Abstract

MiRNAs are key regulators of the mammalian transcriptome that have been increasingly linked to degenerative diseases of the motor neurons. Although many of the miRNAs currently incriminated as participants in the pathogenesis of these diseases are also important to the normal development and function of motor neurons, at present there is no knowledge of the complete miRNA profile of motor neurons. In this review, we examine the current understanding with respect to miRNAs that are specifically required for motor neuron development, function and viability, and provide evidence that these should be considered as a functional network of miRNAs which we have collectively termed MotomiRs. We will also summarize those MotomiRs currently known to be associated with both amyotrophic lateral sclerosis (ALS) and spinal muscular atrophy (SMA), and discuss their potential use as biomarkers.

## Introduction

The expansion of non-coding RNA in higher organisms has been suggested to be a primary determinant of biological complexity (Liu et al., 2013). The conventional understanding is that the majority of the human genome is primarily transcribed into functional non-coding RNA, while only ~2% is dedicated to coding regions (Djebali et al., 2012). Therefore, it has been proposed that the key to understanding human evolution and development lies within the highly dynamic expression of the non-coding genome (Mattick, 2011; Liu et al., 2013; Peschansky and Wahlestedt, 2014).

MicroRNAs (miRNAs) are endogenous small non-coding RNAs which regulate gene expression via formation of a ribonucleoprotein (RNP) complex with Argonaute (AGO) proteins and complementary base pairing with their target mRNAs (Lee et al., 1993; Slack et al., 2000). Despite the apparent simplicity of miRNA function, their expression and mRNA targets are highly dependent on the stage of development, environmental cues, aging and cellular type. This highly dynamic process allows for the fine-tuning of gene expression depending not only on the global needs of the cell, but also somatotopically-specific needs such as the maintenance of the synaptic junction or the response to neuronal injury (van Rooij et al., 2007; Wilczynska and Bushell, 2015).

The discovery that miRNAs are critical to motor neuron function and survival (Haramati et al., 2010) led to the study of the participation of miRNAs in degenerative disorders of motor neurons, most prominently amyotrophic lateral sclerosis (ALS) and spinal muscular atrophy (SMA) type I (Campos-Melo et al., 2013; Ishtiaq et al., 2014; Emde et al., 2015; Luchetti et al., 2015; Murdocca et al., 2016). In both, alterations of miRNA expression have been observed, with ALS showing a profound global dysregulation. However, our understanding of how motor neuron specific miRNAs function synergistically to promote motor neuron survival and how this balance is disrupted in pathological conditions remains incomplete. In this review article, we have examined the evidence for a discrete network of miRNAs that are critical to the biology of motor neurons, which we have termed MotomiRs, and evaluated those dysregulated in motor neuron disease.

## Motor Neurons

Motor neurons are classified as either somatic or visceral. Visceral motor neurons are responsible for innervating smooth and cardiac muscle allowing for involuntary contractions. In contrast, somatic motor neurons innervate skeletal muscle to perform voluntary movement (Goulding, 1998; Guthrie, 2007). This review will focus on somatic motor neurons (henceforth referred to as “motor neurons”) and their overall physiology at the cellular and molecular level.

**Figure 1:**
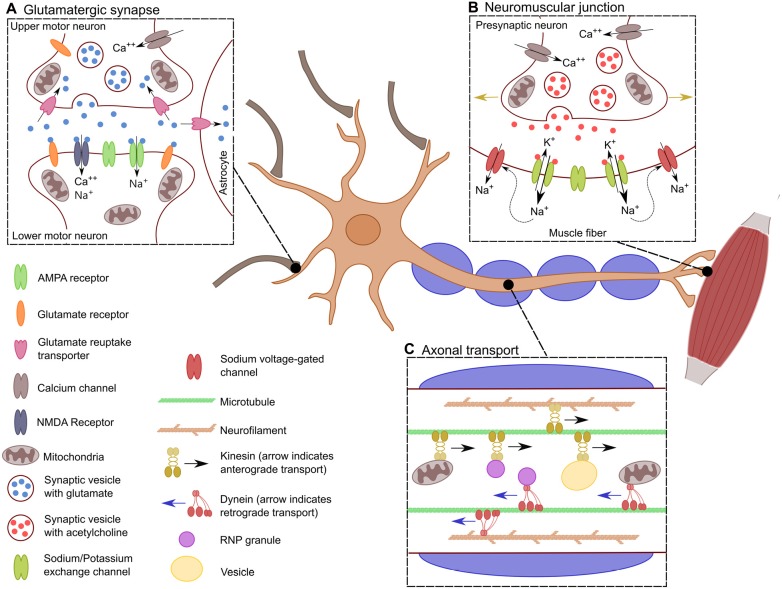
**Specific characteristics of motor neurons**. Schematic showing the distinctive characteristics of motor neurons. **(A)** The glutamatergic synaptic connection between lower motor neurons (LMNs) and upper motor neurons (UMNs). This synapse results in the excitation of the LMN depending of the influx of Ca^++^ into the presynaptic neuron for the release of glutamate into the synaptic cleft. There, glutamate stimulates the influx of Na^+^ and Ca^++^ into the post-synaptic LMN, which leads to its depolarization. **(B)** Transport across the long axon of motor neurons. Neurons must be constantly transferring mitochondria, vesicles and RNPs granules to localized spots in the axon depending on the cells need. Transport is bidirectional along the axon where kinesin allows for anterograde transport, dynein provides retrograde transport. This is crucial for the proper distribution of proteins, transcripts and organelles. **(C)** Neuromuscular junction. When the action potential has reached the neuromuscular junction, there is an influx of Ca^++^ into the axonal bouton resulting in the synaptic release of acetylcholine. This causes the efflux of K^+^ and the influx of Na^+^ leading to the depolarization of the muscle fiber. The influx of Na^+^ causes the opening of sodium voltage-gated channels along the muscle fiber, allowing for the action potential to propagate through the muscle fiber (indicated by the golden arrows), generating the muscle contraction.

In general, motor neurons can be further grouped into those whose projections remain within the central nervous system (CNS) and those which do not. Upper motor neurons (UMNs) are a group of descending supraspinal neurons, the majority of which arise from the primary motor cortex, premotor cortex and the supplementary motor area (Dum and Strick, 2005; Nachev et al., 2008). UMNs arising within these cortical regions course through the corticospinal tracts with the majority (75%–90%) crossing at the level of the medulla to ultimately innervate contralateral spinal motor neurons. The remaining 10%–25% innervate ipsilateral spinal motor neurons (Figure 1A). Spinal motor neurons, collectively termed lower motor neurons (LMNs), in concert with the muscle fibers that they innervate through their axonal terminals, constitute the motor unit (Lemon, 2008; Welniarz et al., 2016; Figure 1B).

### Motor Neuron Development

During development, neural progenitor cells within the ventral region of the neural tube differentiate into either interneurons, glial cells or LMNs (Leber et al., 1990). Differentiation into LMNs is a tightly regulated process governed by the coordinated expression of a number of transcription factors. For example, the expression of transcription factor NK6 homeobox 1 and 2 (Nkx6.1/6.2) in the absence of Iroquois Homeobox 3 (Irx3) and Nkx2.2 expression promotes differentiation into motor neurons (Briscoe et al., 2000). Nkx6.1/6.2 stimulates the expression of oligodendrocyte transcription factor 2 (Olig2), specifically in the motor neural progenitor domain, which then promotes Neurogenin 2 (Ngn2) expression. This in turn leads to cell cycle exit and the induction of motor neuron and pancreas homeobox 1 (Mnx1, also known as Hb9) and choline acetyltransferase (ChAT). Both HB9 and ChAT are key markers of fully differentiated LMNs (Arber et al., 1999; Novitch et al., 2001). LMNs are then further differentiated into lateral, hypaxial, or medial motor columns in response to the expression of either the combination of Forkhead Homeobox 1 (FOXP1)/ISL LIM Homebox 2 (ISL2), HB9/ISL1-2/ETS Variant 1 (ETV1), or HB9/ISL1-2/LIM Homeobox 3 (LHX3), respectively. Generally, differential expression of homeobox (hox) genes plays an essential role in the rostrocaudal and dorsoventral patterning of LMNs within the spinal cord tissue (Guthrie, 2007; Davis-Dusenbery et al., 2014; Stifani, 2014).

Neural progenitor cells within the cortex also express a wide array of genes which results in their differentiation into UMNs. These include *forebrain embryonic zinc finger-like protein 2* (*Fezf2*) and *LIM domain binding 2* (*Ldb2*) which have been shown to determine UMN differentiation, and *COUP-TF1 interacting protein 2* (*Ctip2*) which is critical to promoting axonal growth and guidance (Arlotta et al., 2005; Molyneaux et al., 2005, 2007; Leone et al., 2017). The cascading expression of genes during development is necessary for motor neuron formation, and thus genes must be tightly regulated in a temporal fashion to ensure the right genes are expressed at the right time.

### Axonal Transport

An individual motor neuron axon can extend for upwards of a meter, giving rise to a unique set of metabolic demands. Mitochondria, RNP granules, and vesicles must be positioned at specific sites along the length of the axon depending on somatotopic needs and thus requiring a high degree of regulation (Figure 1C; Vuppalanchi et al., 2009; Lin and Sheng, 2015). Critical to the integrity of the axonal projection are key cytoskeletal proteins, including microtubules which provide the highway that guides axonal transport and neurofilaments which are key to maintaining the cytoskeletal architecture of the axon and thus indirectly, the degree of myelination (Hirokawa et al., 2010; Szaro and Strong, 2010). Further, axons and synapses are highly dynamic structures which are constantly changing in an activity-dependent manner and thus there is a constant redistribution of mitochondria depending on localized energy demands (Miller and Sheetz, 2004). This is especially true for motor neurons as their axonal length requires precise movement of mitochondria to meet their high energy demands (Hinckelmann et al., 2013).

Beyond mitochondrial trafficking, the transport of mRNA along the axon allows for somatotopically-specific protein synthesis. To accommodate this, mRNAs and translational machinery are incorporated into RNP granules and transported to distal regions along the axon for localized translation (Sutton and Schuman, 2006; Vuppalanchi et al., 2009). It is widely accepted that during RNP transport that the mRNA within the granule is translationally silent. This is suggested to be largely mediated by various RNA-binding proteins (Bramham and Wells, 2007). However, it has also been shown that proteins essential for miRNA processing are found within these RNP transport granules (Barbee et al., 2006), suggesting post-transcriptional regulation of mRNA within RNPs is likely an interaction between RNA-binding proteins and regulatory RNA molecules, allowing for localized protein synthesis and even mRNA degradation.

### Regeneration

One of the fundamental differences between mature UMNs and LMNs is the ability for LMNs to regenerate. In general, the peripheral nervous system (PNS) in which LMNs reside provides an environment where neurons can survive and regenerate subsequent to axonal damage. This is not the case in the CNS (Fitch and Silver, 1997; Fu and Gordon, 1997). This phenomenon has been attributed to the lack of neurotrophic factors and an abundance of inhibitory proteins present after a nerve injury in the CNS (Schwab, 1996). For example, the increased expression of neurotrophic factors brain-derived neurotrophic factor (BDNF) and fibroblast growth factor 2 (FGF-2) and their receptors—trkB and FGFR-1, respectively—promotes LMN regeneration in response to a nerve injury. However, within the CNS, the expression of these receptors and ligands are reduced after nerve injury, creating a less permissive environment for regeneration (Funakoshi et al., 1993; Kobayashi et al., 1996; Lewin and Barde, 1996). Interestingly, ectopic expression of BDNF within the CNS after a neuronal injury enhances the regenerative capacity of the neurons, suggesting it is an essential protein for nerve recovery (Giehl and Tetzlaff, 1996; Kobayashi et al., 1997).

Further, the up-regulation of the expression of cytoskeleton proteins including actin, tubulin and peripherin also assist with the regeneration of LMNs by restructuring the axon as it recovers (Bisby and Tetzlaff, 1992; Chadan et al., 1994; Jiang et al., 1994). However, there is a down-regulation in neurofilament expression after a nerve injury, which has been shown to allow for efficient transport of actin, tubulin and peripherin to distal regions of the injured axon (Tetzlaff et al., 1996; Zhu et al., 1998). This change in cytoskeleton proteins after an axonal injury is far less robust within the CNS, and thus could be another reason why regeneration is not able to occur within UMNs (Tetzlaff et al., 1991; Kost and Oblinger, 1993). This tightly coordinated change in the expression of cytoskeleton genes within the PNS allows for efficient axonal repair, and thus, a transient network of regulatory elements, such as miRNAs, must play an essential role in this regenerative process.

## MiRNAs

MiRNAs are evolutionary conserved non-coding RNAs (ncRNAs) of 18–22 nucleotides that post-transcriptionally regulate the expression of most mammalian genes. First discovered in *Caenorhabditis elegans* 20 years ago, miRNAs are the dominant class of small RNAs in somatic cells (Lee et al., 1993; Ha and Kim, 2014). The human genome harbors more than 2500 mature miRNAs that play major roles in a variety of biological pathways such as apoptosis, cell proliferation, development, differentiation and pathological processes.

### Canonical Pathway of miRNA Biogenesis

In mammals, the majority of miRNAs are encoded within introns of either protein-coding or non-coding genes (Rodriguez et al., 2004). Several miRNA loci close in proximity are generally co-transcribed, thus constituting a miRNA cluster. Most miRNAs are transcribed by RNA polymerase II (Pol II); however RNA Pol III has been also shown to transcribe some viral and human miRNAs (Pfeffer et al., 2005; Borchert et al., 2006). MiRNA transcription is controlled by RNA Pol II-associated transcription factors such as MYC ZEB1 and ZEB2 and epigenetic regulators (Cai et al., 2004; Lee et al., 2004; Davis-Dusenbery and Hata, 2010). Transcription products—primary miRNAs that are over 1 kb in length (pri-miRNAs)—contain a stem-loop structure in which mature miRNA sequences are embedded. Similar to mRNAs, pri-miRNA transcripts contain a 7-methyl guanylate cap at the 5′ end and a poly (A) tail at the 3′ end (Davis and Hata, 2009). The nuclear RNAse III-type endonuclease Drosha, and its essential cofactor DiGeorge syndrome chromosomal region 8 (DGCR8), form the microprocessor complex to target and cleave pri-miRNAs at the stem-loop to release the ~65 nt length precursor miRNA (pre-miRNA; Lee et al., 2003; Denli et al., 2004; Gregory et al., 2004; Han et al., 2004). The pre-miRNA is then exported to the cytoplasm by exportin-5 and Ras-related nuclear protein guanosine-5′-triphosphate (Ran-GTP; Yi et al., 2003; Bohnsack et al., 2004; Lund et al., 2004). It has been reported that exportin-5 is necessary but not critical for miRNA maturation, suggesting that other mechanisms complement its function (Kim et al., 2016).

**Figure 2:**
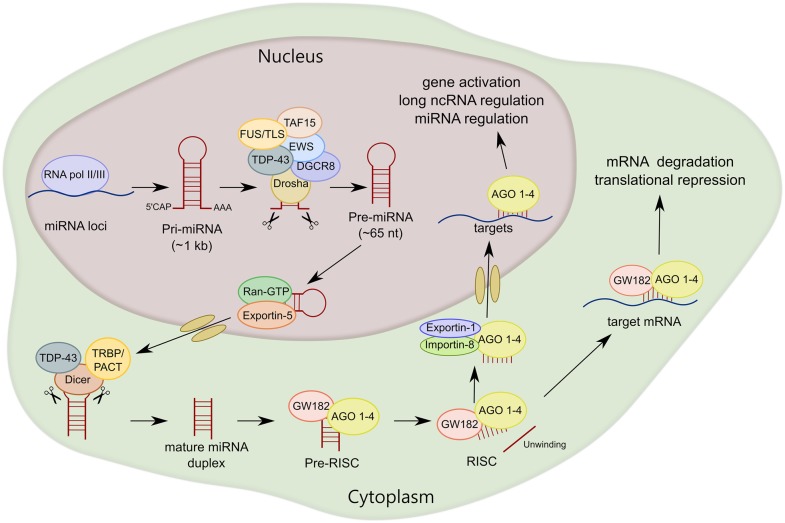
**MiRNA biogenesis of canonical miRNAs**. The first part of miRNA processing occurs in the nucleus. Primary miRNA (pri-miRNA) is transcribed by RNA polymerase II or III (RNA pol II/III) and then cleaved by Drosha/DiGeorge syndrome chromosomal region 8 (DGCR8) to form precursor miRNA (pre-miRNA). Pre-miRNA is exported to the cytoplasm by exportin-5 and then cleaved by Dicer. MiRNA duplex is loaded into argonaute proteins (AGO 1–4) and subsequently unwound into two separated strands. For most miRNA targets, AGO is recruited to a complex that contains GW182 proteins (RNA-induced silencing complex, RISC) that induces translational repression and degradation of the mRNA targets. TAR DNA-Binding Protein 43 (TDP-43) and FET family, RNA-binding proteins linked to amyotrophic lateral sclerosis (ALS), interact with Drosha and/or Dicer, regulating miRNA processing at both primary and precursor levels.

Pre-miRNA is cleaved by another RNAse III-type endonuclease called Dicer, releasing the small miRNA duplex (Hutvágner et al., 2001; Ketting et al., 2001). Dicer associates with the cofactors human immunodeficiency virus transactivating response RNA-binding protein (TRBP) and protein activator of the interferon-induced protein kinase (PACT), which do not seem to be essential for Dicer-mediated pre-miRNA processing (Chendrimada et al., 2005; Haase et al., 2005; Lee et al., 2006, 2013). However, it has been shown that TRBP is an integral cofactor for Dicer processing in RNA-crowded environments, acting as a gatekeeper to preclude Dicer from engaging with pre-miRNA-like substrates (Fareh et al., 2016). After Dicer processing, the miRNA duplex is loaded into AGO proteins (AGO 1–4 in humans) to form the RNA-induced silencing complex (RISC). Subsequently, the miRNA is unwound into two separate strands. The guide strand, which is determined during the AGO loading step based on relative thermodynamic stability, is usually much more prevalent and more biologically active than the passenger strand (miRNA*; Kawamata and Tomari, 2010; Ha and Kim, 2014). After AGO-mature miRNA binding, AGO seeks target mRNAs that are complementary to the miRNA seed sequence. Of note, miRNA silencing likely occurs by submicroscopic complexes in the cytoplasm that are constantly exchanging with cytoplasmic RNA granules called P-bodies (Leung and Sharp, 2013). Finally, an interesting observation is that most mature miRNAs are also present in the nucleus, indicating that mature miRNAs can shuttle between the nucleus and cytoplasm. Exportin-1 and importin-8 have been shown to mediate the translocation to the nucleus of not only miRNAs, but also AGO proteins (Castanotto et al., 2009; Weinmann et al., 2009). Within the nucleus, miRNAs can function in gene activation, or in an unconventional manner regulating the biogenesis and functions of miRNAs and long ncRNAs (Liang et al., 2013; Figure 2).

### Non-Canonical Pathways of miRNA Biogenesis

Several alternative mechanisms of miRNA biogenesis have been described besides the canonical pathway, although only about 1% of conserved miRNAs are produced independently of Dicer or Drosha in vertebrates (Ha and Kim, 2014). The most common non-canonical pathway is used for mirtron production: miRNAs encoded in introns at the exon junction site. Mirtron miRNAs bypass the Drosha-DGCR8 complex. Pre-miRNAs are instead generated by mRNA splicing, lariat debranching and trimming (Berezikov et al., 2007; Ruby et al., 2007). Drosha-mediated processing is also bypassed in the case of miRNAs derived from shRNAs, tRNAs or tRNA-like precursors, small nucleolar RNAs (snoRNAs) or snoRNA-like viral RNAs (Babiarz et al., 2008; Ender et al., 2008; Cazalla et al., 2011).

In some cases, such as miR-451, miRNAs can also be generated by Dicer independent miRNA biogenesis. After Drosha cleavage, pre-miR-451 is directly loaded and sliced by AGO2 (Cifuentes et al., 2010). Then, a poly(A)-specific ribonuclease (PARN) trims down the 3′ end of pre-miR-451 to produce the mature miR-451 (Yoda et al., 2013).

**Figure 3:**
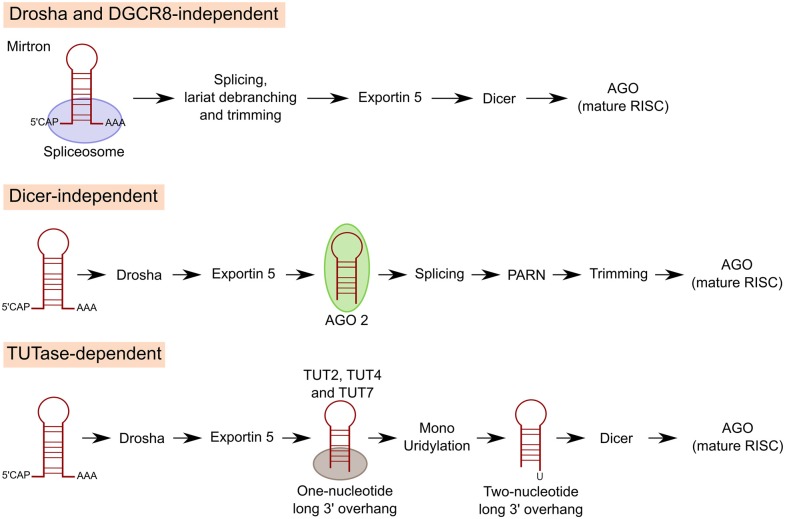
**Non-canonical pathways of miRNA processing**. For mirtron production, miRNAs encoded in introns at the exon junction site, miRNAs bypass Drosha/DGCR8 and pre-miRNAs are instead generated by mRNA splicing, lariat debranching and trimming. In other cases, miRNAs can also be generated by Dicer independent miRNA biogenesis. After Drosha cleavage miRNA is directly loaded and sliced by AGO2. Then, poly(A)-specific ribonuclease (PARN) trims down the 3′ end of pre-miRNA to produce the mature miRNA. A third class of miRNAs undergo Drosha and Dicer dependent biogenesis but an additional processing step is included in between the two RNAses. Precursors of these miRNAs carry a shorter 3′ overhang. Terminal uridylyl transferases (TUT2, TUT4 and TUT7) target these pre-miRNAs and extend their 3′ end by 1 nucleotide through monouridylation for efficient Dicer processing.

Another class of miRNAs undergo Drosha and Dicer dependent biogenesis but an additional processing step is included in between the two RNases. Precursors of these miRNAs carry a shorter (one-nucleotide long instead of two) 3′ overhang. Terminal uridylyl transferases (TUT2, TUT4 and TUT7) target these pre-miRNAs and extend their 3′ end by one nucleotide through monouridylation for efficient Dicer processing (Heo et al., 2012; Figure 3). Of interest, TUTs can also trigger pre-miRNA degradation through oligouridylation of 3′ trimmed pre-miRNAs and pre-let-7 (see “Regulation of miRNA Expression” Section; Kim et al., 2015).

### Regulation of miRNA Expression

MiRNA expression can be regulated at multiple levels. Transcription is the first control point of the miRNA biogenesis. Of note, one-third of intronic miRNAs have transcription initiation regions independent of their host promoters. RNA Pol II-transcribed miRNA promoters are generally similar to mRNA promoters in terms of frequencies of CpG islands, TATA elements, TFIIB recognition elements, initiator elements (Inr), motif ten elements (MTE) and downstream promoter elements (DPE). Also, some transcription factors that control mRNA production regulate the transcription of miRNAs encoded in introns of protein coding genes (Ozsolak et al., 2008; Davis and Hata, 2009).

Changes in the methylation of miRNA promoters or miRNA sequences can impact on the expression of miRNAs. The methylation status of some miRNA genes has also been suggested to be key to the pathogenies of certain cancers. For example, hypermethylation of the CpG island upstream of the tumor suppressor miR-33b, is responsible for its down-regulation in gastric cancer (Yin et al., 2016). Methylation of promoters of the miR-200b cluster is associated with metastasis in advanced breast cancer (Wee et al., 2012). Interestingly, methylation of the 5′ monophosphate of pre-miR-23b and pre-miR-145 inhibits the processing of these miRNAs by Dicer (Xhemalce et al., 2012). This is because the interaction between Dicer and the 5′ monophosphate is necessary for the efficient processing of pre-miRNAs (Park et al., 2011). MiRNA promoters are also regulated by histone modifications. Some miRNAs have been reported to be up- or down-regulated after the treatment with histone deacetylase (HDAC) inhibitors (Saito and Jones, 2006; Scott et al., 2006; Nasser et al., 2008). For instance, acetylation regulates the expression of miR-133a during chronic pressure overload-induced cardiac fibrosis (Renaud et al., 2015). Single nucleotide polymorphisms (SNPs) in miRNA genes can also affect miRNA biogenesis (Duan et al., 2007). For example, SNPs in miR-1206 and miR-612 genes within two cancer risk loci affect the expression of both mature miRNAs (Kim et al., 2012).

RNA editing (adenosine to inosine catalyzed by adenosine deaminase that acts on RNA; ADARs) also impacts on miRNA processing. Let-7 pri-miRNA editing impairs the biogenesis of this miRNA and drives leukemia stem cell self-renewal (Zipeto et al., 2016). Another type of regulation of miRNA biogenesis is by RNA-tailing (nucleotidyl addition to the 3′ end of RNA). For example, LIN28 proteins recruit terminal uridylyl transferases TUT4 and TUT7 C6 to induce oligouridylation of pre-let-7 (Heo et al., 2008, 2009; Hagan et al., 2009). This oligo-U tail blocks Dicer processing, and facilitates miRNA decay by 3′-5′ exonuclease DIS3L2 (Chang et al., 2013; Ustianenko et al., 2013). Some miRNA transcripts, like most mRNAs, are methylated at N6-adenosine (m6A). This modification acts as a mark for pri-miRNA processing. RNA-binding protein hnRNPA2B1 binds to m6A, interacts with DGCR8, and promotes pri-miRNA cleavage to produce pre-miRNA (Alarcón et al., 2015). Finally, levels of certain miRNAs are controlled by regulating miRNA stability. For instance, levels of miR-122 are stabilized by monoadenylation via the non-canonical cytoplasmic poly(A) polymerase GLD-2 (TUT2) in mammals (Katoh et al., 2009; D’Ambrogio et al., 2012). Moreover, a highly complementary mRNA target can induce miRNA degradation through 3′ addition of a single non-templated uridine followed by 3′ to 5′ trimming of the miRNA with a 2′-O-methyl group added by Hen1 enzyme in *Drosophila* (Ameres et al., 2010; Baccarini et al., 2011).

Recently, another layer of complexity has been added into miRNA regulation. Levels of mature forms of miR-122 are post transcriptionally regulated by modulating its processing in a target-dependent manner during recovery from starvation-related stress (Bose and Bhattacharyya, 2016).

### Mechanisms of Action of miRNAs

**Figure 4:**
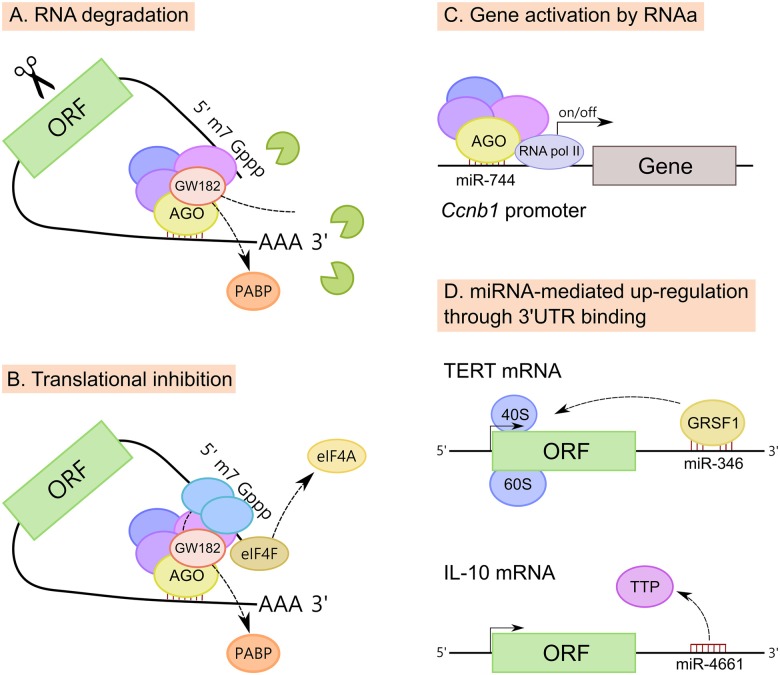
**Mechanisms of action of miRNAs. (A)** MiRNAs promote mRNA degradation by recruiting deadenylases on the target mRNA via GW182 and also through the dissociation of PABP, increasing the accessibility of the poly(A) tail to deadenylases. **(B)** MiRNAs inhibit translation at the initiation step, however the exact mechanism is still unclear. Three mechanisms have been proposed; (i) PABP displacement mediated by GW182; (ii) recruitment of the translational repressors through GW182; and (iii) dissociation of eukaryotic initiation factor-4A (eIF4A) from the cap-binding complex eIF4F. **(C)** MiRNAs also induce up-regulation of their targets. MiRNAs have been implicated in gene activation triggered by promoter-targeted small RNAs, known as RNA activation (RNAa). **(D)** Up-regulation of certain transcripts can also be mediated by miRNA binding to mRNA 3′untranslated regions (3′UTRs), resulting in either translation activation or RNA stability enhancement. MiR-346-dependent up-regulation of telomerase reverse transcriptase (TERT) occurs through the binding to TERT mRNA 3′UTR and is mediated by G-rich RNA sequence binding factor 1 (GRSF1). MiR-346 facilitates the recruitment of TERT mRNA to ribosomes to promote translation. In another example, miR-4661 uses the binding sites of the tristetraprolin (TTP) in the IL-10 3′UTR preventing TTP-mediated IL-10 mRNA degradation in macrophages.

MiRNAs are able to regulate gene expression by several mechanisms (Figure 4). In RNA silencing, miRNAs function as a guide to recognize target mRNAs, whereas AGO proteins function as effectors by recruiting factors that induce translational repression and/or mRNA decay (Ha and Kim, 2014). The 5′ region of the miRNA (seed, nucleotides 2–8) is crucial for target recognition through the complementary base pairing of miRNA recognition elements (MREs) that are mostly localized in the mRNA 3′untranslated region (UTR). Currently, MREs in 3′UTRs are determined using prediction algorithms and then validated with functional analysis. However, seedless 3′UTR MREs have been described, as well as MREs localized within 5′UTRs and coding regions (Forman et al., 2008; Lal et al., 2009; Moretti et al., 2010). To provide an alternative view of human miRNA targets, a protocol termed cross-linking, ligation and sequencing of hybrids (CLASH) was developed for high-throughput identification of miRNA-target RNA duplexes associated with AGO, and is independent of bioinformatic predictors. Transcriptome-wide data set revealed that binding of most miRNAs to their targets includes the seed region, but around 60% of seed interactions are noncanonical. Of interest, seed interactions are generally accompanied by non-seed base pairing (Helwak et al., 2013).

MiRNAs down-regulate target mRNAs through translational repression and mRNA destabilization (Figures 4A,B), with mRNA destabilization dominating most miRNA-mediated repression (Hendrickson et al., 2009; Guo et al., 2010; Eichhorn et al., 2014). Although miRNAs inhibit translation at the initiation step, the exact mechanism is still unclear. Three mechanisms have been proposed: (i) PABP displacement mediated by GW182 (Moretti et al., 2012; Zekri et al., 2013); (ii) recruitment of the translational repressors through GW182 (Meijer et al., 2013; Kamenska et al., 2014; Waghray et al., 2015); and (iii) dissociation of eukaryotic initiation factor-4A (eIF4A) from the cap-binding complex eIF4F (Fukao et al., 2014; Fukaya et al., 2014).

At the same time, mRNA destabilization is a consequence of miRNA-mediated deadenylation of target mRNAs which causes these mRNAs to undergo decapping and 5′–3′ decay (Rehwinkel et al., 2005; Behm-Ansmant et al., 2006; Wu et al., 2006). MiRNAs promote mRNA decay by recruiting the deadenylase complex CCR4–NOT or PAN2–PAN3 on the target mRNA via GW182 (Braun et al., 2011; Fabian et al., 2011). Also, miRNAs promote mRNA decay through the dissociation of PABP, increasing the accessibility of the poly(A) tail to deadenylases (Moretti et al., 2012).

Beside well-known down-regulatory functions, there is increasing evidence that miRNAs can also induce up-regulation of their targets (Vasudevan et al., 2007; Campos-Melo et al., 2014; Valinezhad Orang et al., 2014). MiRNAs, similar to double stranded RNAs (dsRNAs), have been implicated in gene activation triggered by promoter-targeted small RNAs, known as RNA activation (RNAa; Figure 4C). For instance, the expression of *cyclin B1* (*Ccnb1*) depends on RNAa by miRNAs and components of miRNA biogenesis in mouse cells. Chromatin immunoprecipitation (ChIP) analysis had shown that AGO1 is selectively associated to the *Ccnb1* promoter and miR-744, which induces *Ccnb1* expression, increases enrichment of RNA Pol II and trimethylation of histone 3 at lysine 4 (H3K4me3) at the *Ccnb1* transcription start site. At a functional level, short-term expression of miR-744 enhances cell proliferation, but prolonged overexpression causes tumor suppression (Huang et al., 2012).

Finally, the up-regulation of certain mRNA transcripts can also be mediated by miRNA binding to mRNA 3′UTRs, resulting in either translation activation or RNA stability enhancement (Figure 4D). For example, miR-346-dependent up-regulation of telomerase reverse transcriptase (TERT) occurs through the binding of miR-346 to TERT mRNA 3′UTR. When miR-346 is bound to the TERT mRNA 3′UTR, its middle sequence motif forms a “bulge loop”, facilitating the G-rich RNA sequence binding factor 1 (GRSF1)-mediated recruitment of TERT mRNA to polysomes to promote translation (Song et al., 2015). A similar mechanism of GRSF1 interaction with AGO2 in a miR-346-dependant manner, leading to up-regulate the expression of AGO2, has been described for cervical cancer (Guo et al., 2015). It has also been reported that miR-466l uses the binding sites of the RNA-binding protein tristetraprolin (TTP) in the *IL-10* 3′UTR AU-rich elements, thus preventing TTP-mediated IL-10 mRNA degradation in macrophages (Ma et al., 2010).

## MiRNAs in Motor Neuron Function: MotomiRs

Transgenic mouse models containing loss of Dicer function set the foundation for the importance of miRNA regulation within motor neurons. These experiments showed that in early development, loss of Dicer function within motor neuron progenitor cells leads to aberrant motor neuron development in the lateral motor column, while in adult mice, loss of Dicer expression in motor neurons resulted in progressive motor neurodegeneration (Haramati et al., 2010; Chen and Wichterle, 2012). With these two studies, it became apparent that the production of miRNAs is a critical factor to overall motor neuron function and survival. There is very little known about the miRNome (the full spectrum of miRNAs being expressed) of motor neurons. However, as we further discover miRNAs related to motor neuron development and degeneration, it becomes increasingly evident that there are specific miRNAs needed for motor neuron viability (Table 1). In the following section, we will review the known miRNAs associated with motor neuron homeostasis.

**Table 1:** **List of current MotomiRs and their function**.

MotomiR	Genes shown to regulate	Role within motor neurons	Organism/Cell models used to describe function	References
**miR-9**	*OC1, FoxP1, NEFH, MAP1B, MCPIP1*	Development, cytoskeleton maintenance, cell survival	Chick, mouse neuronal precursor cells, mouse	Haramati et al. (2010), Otaegi et al. (2011), Dajas-Bailador et al. (2012), Luxenhofer et al. (2014) and Xu et al. (2016)
miR-124	*REST, Stat3, Kfl6*	Development, regeneration	Mouse	Visvanathan et al. (2007) and Nagata et al. (2014)
miR-146a*, miR-524, miR-582, miR-b1336, and miR-b2403	*NEFL*	Cytoskeleton maintenance	*In vitro* interactions done in HEK293T cells	Campos-Melo et al. (2013) and Ishtiaq et al. (2014)
miR-218	*Tead1, Foxp2, Lhx1, Slc6a1, Bcl11a, SLC1A1*	Development, membrane excitability, NMJ synaptic connections	Mouse	Amin et al. (2015) and Thiebes et al. (2015)
miR-8	*FasIII, Nrg, wg, lar*	Synaptic plasticity	*Drosophila*	Nesler et al. (2013) and Lu et al. (2014)
miR-958 and miR-289	*lar*	Synaptic plasticity	*Drosophila*	Nesler et al. (2013)
**miR-375**	*PAX6, CCND2, p53*	Development, cell survival	Human neural progenitor cell cultures	Bhinge et al. (2016)
miR-310–313	*Khc-43*	Synaptic vesicle release	*Drosophila*	Tsurudome et al. (2010)
miR-128 and miR-20a	*PDZ-RhoGEF*	Axonal growth, regeneration	Rat cortical neuron cultures	Sun et al. (2013)
miR-153	*SNAP-25*	Axonal growth, synaptic vesicle release	Zebrafish	Wei et al. (2013)
miR-196	*Hoxb8*	Development	*Drosophila*	Asli and Kessel (2010)
**miR-183**	*mTOR*	Neurite growth	Rat primary spinal motor neuron cultures	Kye et al. (2014)
**miR-206**	*BDNF, HDAC4*	NMJ Regeneratoin	Mouse	Williams et al. (2009) and Miura et al. (2012)

*In bold: miRNAs related to amyotrophic lateral sclerosis (ALS) and spinal muscular atrophy (SMA)*.

### Motor Neuron Differentiation

As previously described, motor neuron development is a highly dynamic process that requires precise expression of particular genes at the right time. MiRNAs play an essential role in the temporal expression of genes during motor neuron differentiation. For example, activation of miR-9 within the developing chick results in the repression of the transcription factor onecut1 (OC1), which helps to drive differentiation of neural progenitor cells into spinal motor neurons (Luxenhofer et al., 2014). However, the activation of miR-9 not only leads to spinal motor neuron differentiation, but sustained expression of miR-9 later in development specifies a subset of motor neurons into the medial motor neuron column via suppression of Forkhead Box P1 (FoxP1; Otaegi et al., 2011). Thus, miR-9 is needed for both spinal motor neuron differentiation and localization. Beyond differentiation, miR-9 has also been shown to regulate axonal development within mouse models through the suppression of microtubule associated protein 1B (MAP1B; Dajas-Bailador et al., 2012).

Although miR-9 is crucial for motor neuron specificity during development, other miRNAs have been reported to participate in the process. MiR-218 is activated upon the co-expression of motor neuron specific transcription factors Isl1 and Lhx3, and directly suppresses *TEA Domain Transcription Factor 1* (*Tead1*), *Solute Carrier Family 6 Member 2* (*Slc6a1), B-cell lymphoma/leukemia 11A* (*Bcl11a)*, *Lhx1*, *Paired box 2* (*Pax2)* and *FoxP2* (Thiebes et al., 2015). Suppression of the latter three genes diverts neural progenitor cells away from interneuron differentiation and towards motor neuron differentiation. However, miR-218 expression alone cannot push neural progenitor cells towards motor neuron differentiation; therefore, miR-218 must be working in concert with other miRNAs or other regulatory molecules to establish motor neuron identity (Thiebes et al., 2015).

While the activation of Isl1 and Lhx3 transcription factors is required for the expression of motor neuron specific miRNAs, inhibition of RE1-silencing transcription factor (REST) is also required to promote motor neuron identity via activation of miR-375 (Bhinge et al., 2016). REST silencing has been shown to be mediated by miR-124 during development to promote neurogenesis (Visvanathan et al., 2007). Once REST is silenced, as demonstrated in REST knock-out mice, there is an increase in the expression of miR-375. MiR-375 directly targets *PAX6* and *Cyclin D2* (*CCND2*) transcripts, silencing their expression which in turn promotes motor neuron differentiation (Bhinge et al., 2016). However, PAX6 is also necessary for the production of neural progenitor cells (Bel-Vialar et al., 2007). Thus, miR-124 must silence REST activity in a timely manner to promote the expression of miR-375, which in turn, must also suppress PAX6 activity in a timely manner to allow for the formation of post-mitotic spinal motor neurons (Visvanathan et al., 2007; Bhinge et al., 2016).

Beyond regulating PAX6 and CCND2 levels, miR-375 also reduces tumor suppressor p53 protein levels. This interaction is considered necessary for motor neuron survival as p53 is a pro-apoptotic gene (Bhinge et al., 2016). DNA damage in developing motor neurons leads to an increased expression of p53, which ultimately results in programmed cell death (Lavin and Gueven, 2006). Conversely, overexpression of miR-375 can prevent cell death within developing motor neurons even after a cell has experienced DNA damage (Bhinge et al., 2016). Thus, not only is miR-375 needed for motor neuron differentiation, but sustained expression of this miRNA appears to be critical for preventing apoptosis via inhibition of p53.

As previously mentioned, *Hox* genes play an essential role in the spatial patterning of neurons within both brain and spinal cord tissue, and thus it is not surprising that miRNAs are involved in the precise regulation of *Hox* genes in a spatial and temporal manner during development (Mallo and Alonso, 2013). In particular, expression of miR-196 at a specific time during development is required for proper motor neuron differentiation in chick embryos via down-regulation of *Homeobox B8* (*Hoxb8*). Failure to clear Hoxb8 in a spatial and temporal manner leads to the abolition of motor neuron genesis within the chick neural tube leaving cells in a neural progenitor cell state. However, the inhibition of miR-196 alone does not lead to sustained expression of Hoxb8 throughout the neural tube as might be expected, suggesting other miRNAs or regulatory elements are working in concert with miR-196 to silence Hoxb8 expression and promote motor neuron development (Asli and Kessel, 2010).

### Cytoskeletal Integrity

Neurofilaments are the main cytoskeletal intermediate filaments. Described on the basis of their molecular weights, the individual neurofilament subunit proteins (low, medium and high molecular weight neurofilament subunits; NFL, NFM and NFH, respectively) must maintain a specific stoichiometry (Carpenter and Ip, 1996). The loss of this specific stoichiometry leads to neurofilament aggregation, loss of axonal integrity and neuronal apoptosis (Szaro and Strong, 2010). While the full complement of miRNAs involved in the regulation of neurofilament expression remains to be characterized, it is known that miR-9 regulates the expression of *NEFH* mRNA (Haramati et al., 2010). Reduced levels of miR-9 within rodents has been linked to the loss of *NEFH* mRNA suppression and motor neuron death (Haramati et al., 2010). Our group subsequently demonstrated that miR-146a*, miR-524-5p, miR-582, miR-b1336 and miR-b2403 are critical regulators of *NEFL* expression (Campos-Melo et al., 2013; Ishtiaq et al., 2014). We studied those miRNAs capable of destabilizing *NEFL* mRNA which are up-regulated in ALS spinal cord, as well as those miRNAs capable of stabilizing *NEFL* mRNA that are down-regulated. We described a pool of miRNAs, and by inference, the *NEFL* miRNA network, that is altered in a manner which would be predicted to disrupt neurofilament stoichiometry in ALS motor neurons.

### NMJ Function and Plasticity

In *Drosophila* models, miR-8 is the most widely studied miRNA in reference to the NMJ. At the NMJ, miR-8 was first suggested to play an essential role in NMJ connectivity and expansion by limiting the post-synaptic expression of a highly-conserved actin regulatory protein—Enabled (Ena). This affect by miR-8 reduces actin polymerization on the post-synaptic terminal, and creates presynaptic arbors optimizing bouton connectivity (Loya et al., 2009, 2014).

MiR-8 is also involved in regulating cell adhesion molecules (CAMs) at both the pre- and post-synaptic membranes in order to coordinate synaptic connections at the NMJ (Lu et al., 2014). Post-synaptically, miR-8 regulates Fasciclin III (FasIII) while the pre-synaptic expression of miR-8 regulates Neuroglian (Nrg) expression. While miR-8 has no direct target within the 3′UTR of *FasIII* or *Nrg* transcripts, the deletion of miR-8 leads to decrease levels of these two CAMs (Lu et al., 2014). This suggests that miR-8 likely targets and silences upstream transcripts that negatively regulate CAM molecules to produce strong synaptic connections at the NMJ.

Consistent with this theory, Nesler et al. (2013) showed that five miRNAs acted in an activity-dependent manner at the NMJ within *Drosophila* larvae, including miR-8. Further, the authors showed that miR-8 directly targets and supresses Wingless (Wg) expression which is an essential gene for NMJ development and plasticity, while miR-8, along with miR-289 and miR-958, silenced Leukocyte-antigen-related-like (Lar) expression, which is important for synaptic growth and motor axonal extension (Kaufmann et al., 2002; Koles and Budnik, 2012; Nesler et al., 2013). This suggests that miR-8, miR-289, and miR-958 are crucial for motor connectivity and plasticity at the NMJ in *Drosophila* through the regulation of Wg and Lar. Further, loss of neuronal expression of miR-8, miR-289, and miR-958 reduced synaptic growth at the NMJ in an activity-dependent manner (Nesler et al., 2013). While the latter study also discussed miR-1 and miR-314 as miRNAs that change expression in an activity-dependent manner at the NMJ, the loss of these two miRNAs had no effect on NMJ activity.

MiRNA cluster (miR-310/313) has also been shown to be necessary for NMJ function (Tsurudome et al., 2010). In *Drosophila* larva, the loss of miR-310/313 induces the enhancement of excitatory synaptic release, which can be reversed by the re-introduction of miR-310/313 cluster. MiR-310/313 regulates a signal transduction pathway by directly targeting and silencing *kinesin heavy chain 43* (*Khc-43)* mRNA, leading to the indirect regulation of Bruchpilot (Brp) and Cacophony (Cac) proteins. Khc-43 promotes Brp expression which in turn localizes Cac to the NMJ, promoting calcium influx and acetylcholine release (Tsurudome et al., 2010). Hence, the regulation of the Khc-43 pathway via miR-310/313 is critical to provide control of muscle activation at the NMJ.

Another miRNA involved with NMJ neurotransmitter release is miR-153. The motor system within zebrafish requires precise regulation of synaptosome associated protein 25 (SNAP-25), which is required for neurotransmitter release (Wei et al., 2013). *SNAP-25* expression is post-transcriptionally suppressed by miR-153 specifically within motor neurons, which in turn reduces synaptic vesicle release to regulate muscle activation (Wei et al., 2013).

The motor neuron specific miRNA, miR-218, has been predicted to target and suppress 333 mRNA molecules (termed TARGET^218^) within the motor neuron transcriptome to elicit and maintain neuromuscular synapses, membrane excitability and motor neuron survival. Of note, *solute carrier family 1 member 2* (*Slc1a2*) mRNA, a glutamate re-uptake receptor, was the most up-regulated gene after miR-218 suppression (Amin et al., 2015), suggesting this miRNA may play an essential role in preventing neuronal excitoxicity—a major contributor to motor neurodegeneration.

Finally, miR-206 is a skeletal muscle specific miRNA which plays an essential role in NMJ maintenance and regeneration (Ma et al., 2015). After an acute nerve injury in wild-type mice, the up-regulation of miR-206 suppresses *HDAC4* mRNA expression via direct interactions with the 3′UTR, and promotes the expression of fibroblast growth factor binding protein (FGFBP1; Williams et al., 2009). Subsequently, FGFBP1 is released into the extracellular matrix to potentiate FGF-7 bioactivity, which acts on the distal axon to promote re-innervation (Beer et al., 2005). Others have also described miR-206 to be a negative regulator of BDNF within skeletal muscle (Miura et al., 2012; Ma et al., 2015). This is intriguing, as despite the fact that miR-206 has been described as a skeletal muscle specific miRNA, it has also be shown to be expressed within spinal motor neurons (Chakrabarti et al., 2014). As previously mentioned, expression of FGFs and BDNF in motor neurons are necessary components of regeneration, and thus one could speculate that miR-206 likely regulates the expression of BDNF and FGFs within spinal motor neurons to facilitate neurotrophic signaling between muscle fibers and motor axons after an acute nerve injury.

### Regeneration

Both miR-9 and miR-124 are highly enriched within neurons in the human brain and spinal cord tissue, suggesting that they are critical for neuronal function. However, during motor neuron regeneration there is a shift in the expression of these two miRNAs. For example, during motor neuron injury miR-124 is suppressed, which allows for the up-regulation of *signal transducer and activation of transcription 3* (*Stat3*) and *Kruppel like factor 6* (*Klf6*)—two genes crucial for motor neuron regeneration (Nagata et al., 2014). Further, miR-9 has been shown to be down-regulated in the early stages of a nerve injury, but up-regulated in the later stages. This expression pattern of miR-9 is inversely correlated with *zinc finger CCCH-Type containing 12A* (*MCPIP1*) expression—a pro-apoptotic gene (Xu et al., 2016). The initial down-regulation of miR-9 is considered necessary to initiate cell regeneration, but in order for the cell to regenerate, miR-9 must be up-regulated to prevent the expression of pro-apoptotic genes, otherwise programmed cell death will be initiated. Studies with both miR-9 and -124 suggest a shift in miRNA expression must be made constantly depending on the need of the cell to maintain homeostasis. Therefore, miRNAs needed to sustain motor neuron function may not be the same miRNAs needed for motor neuron recovery, emphasizing the highly dynamic network of miRNA gene regulation.

MiR-128 and miR-20a also appear to be necessary for the development and regeneration of PNS neurons, including motor neurons (Sun et al., 2013). Heat shock protein family B member 1 (HspB1) expression has been shown to be enhanced during neuronal regeneration, while the Ras homolog family member A (RhoA) pathway is suppressed which is considered necessary for neuronal recovery (Fournier et al., 2003; Ma et al., 2011). *In vitro* studies within rat cortical neurons have shown that in order to promote neurite growth, HspB1 must reduce RhoA activity by promoting the expression of miR-128 and miR-20a. These two miRNAs do not interact with *RhoA* mRNA directly, rather they target *Rho Guanine Nucleotide Exchange Factor 11* (*PDZ-RhoGEF*) leading to its suppression. The silencing of *PDZ-RhoGEF* via miR-128 and miR-20a reduces RhoA activity, and hence promotes neurite growth (Sun et al., 2013). The authors concluded that this pathway is critical for neuronal development, but further suggested that these miRNAs may be necessary for neuronal regeneration as the inverse correlation of HspB1 and RhoA activity is also seen in regenerating motor neurons (Ma et al., 2011; Huelsenbeck et al., 2012).

Beyond the RhoA pathway, mechanistic target of rapamycin (mTOR) is a serine/threonine protein kinase which is functionally a part of two complexes (mTOR complex 1 [mTORC1] and mTOR complex 2 [mTORC2]), and has been described in several cellular processes including axonal regeneration (Berry et al., 2016). Key regulators of the mTOR pathway include miR-183 which directly supresses *mTOR* expression, and even regulates the ratio of mTORC1/2 complexes. The overexpression of miR-183 inhibits neurite outgrowth via regulation of the mTOR pathway (Kye et al., 2014). While it has not been determined if miR-183 basal levels are required for motor neuron survival, mTOR’s involvement in the maintenance of the cellular cytoskeleton and mitochondrial biogenesis/removal would suggest precise regulation of this pathway is likely crucial (Loewith et al., 2002; Zhu et al., 2013). Therefore, the overexpression of miR-183 maybe toxic to motor neurons, but based on what we know about the mTOR pathway, basal levels and/or transient expression of miR-183 might be crucial for overall motor neuron maintenance and cellular regeneration.

### MotomiRs

Several miRNAs have been implicated as being critical for motor neuron development, maintenance, regeneration and survival; which we have termed MotomiRs. Among the MotomiRs, only miR-218 has been described as a motor neuron specific miRNA. MiR-218 is predicted to regulate a large group of genes within the motor neuron transcriptome and its involvement in several motor pathways shows how critical this miRNA is to motor function (Amin et al., 2015; Thiebes et al., 2015). Despite miR-218 being the only described motor neuron specific miRNA, other miRNAs may have motor neuron specific functions. The miRNA regulatory network is a highly dynamic system from cell to cell, as the same miRNA might target different genes between two cell lines depending on the transcripts being expressed. Thus, if we are ever to appreciate the complexity of this highly dynamic network in motor neurons, it will be critical to understand its miRNome, their targets, and what specific networks they regulate (Berezikov, 2011). Further, we will have to understand the transient nature of miRNAs within motor neurons (Pothof and van Gent, 2011); when and where are they expressed, and what their expression patterns are at different time periods (e.g., age differences, stress vs. basal conditions, developing vs. mature cells, excited vs. unexcited neurons, health vs. disease, etc.). Answering these questions will be essential in understanding the spatiotemporal expression patterns of MotomiRs, which transcripts are targeted at a particular time and finally how changes to this complex MotomiR network leads to disease.

## MiRNAs in Motor Neuron Diseases: Dysregulation, Diagnosis and Therapy

Given the pivotal roles of miRNAs in regulating motor neuron differentiation, structure, activity and cytoskeletal integrity, it is not surprising that alterations in the expression of miRNAs have been increasingly linked to human motor neuron degenerative disorders. These alterations can be in the miRNAs and/or MREs such as changes in the expression, editing and methylation, mutations and SNPs, and also alterations in competing endogenous RNAs (ceRNAs) involved in the regulation of the interaction of miRNAs and their targets.

### Spinal Muscular Atrophy (SMA)

SMA is an autosomal recessive disease characterized by progressive loss of lower motor neurons and atrophy of muscle (Burghes and Beattie, 2009). Proximal SMA has an incidence of ~1:10,000 newborns and is the most frequent SMA type (Wirth et al., 2006). SMA is caused by homozygous deletion or mutation of *survival motor neuron 1* (*SMN1, Lefebvre et al., 1995*). SMN has been found to play roles in RNA metabolism, specifically in small nuclear RNP (snRNP) biogenesis, alternative splicing, trafficking of RNA-binding proteins and translation of target mRNAs in neurites. SMN also binds to fragile X mental retardation protein (FMRP), KH-type splicing regulatory protein (KSRP) and fused in sarcoma/translocated in liposarcoma (FUS/TLS), which are important for miRNA biogenesis and function (Gubitz et al., 2004; Piazzon et al., 2008; Tadesse et al., 2008; Trabucchi et al., 2009; Akten et al., 2011; Fallini et al., 2011, 2014; Hubers et al., 2011; Yamazaki et al., 2012).

In fact, several lines of evidence have involved miRNAs in SMA. It was reported that mice lacking the miRNA-processing enzyme Dicer selectively in motor neurons display hallmarks of SMA (Haramati et al., 2010). Also, SMN protein has been shown to alter miRNA expression and distribution in neurons (Kye et al., 2014; Wang et al., 2014). Specifically, miR-183 is increased in neurites of SMN-deficient neurons. Inhibition of miR-183 expression in the spinal cord of an SMA mouse prolongs survival and improves motor function of *Smn*-mutant mice (Kye et al., 2014). SMN protein also down-regulates the expression of miR-9a. Interestingly, miR-9a levels have shown a positive correlation with SMA severity (Wang et al., 2014). A more recent study has shown that miR-431, involved in motor neuron neurite length, also plays a role in the SMA motor neuron phenotype. By integrating miRNA:mRNA profiles, it was observed that miR-431 expression is highly increased in spinal motor neurons and a number of its putative mRNA targets are significantly down-regulated in motor neurons after SMN loss (Wertz et al., 2016). Another miRNA involved in SMA motor neuron phenotype is miR-375. Besides its role in neurogenesis, miR-375 protects neurons from apoptosis in response to DNA damage. Motor neurons from a SMA patient have shown reduced levels of miR-375, elevated p53 protein levels, and higher susceptibility to DNA damage induced apoptosis (Bhinge et al., 2016).

Recently, the first vertebrate system allowing transgenic spatio-temporal control of the *smn1* gene was developed using stable miR-mediated knockdown technology in zebrafish. The expression of anti-*smn1* miRNAs in motor neurons reproduced most hallmarks observed previously in the ubiquitous knockdown model. In addition, *smn1* knockdown in zebrafish motor neurons is sufficient to induce late-onset motor neuron degeneration (Laird et al., 2016). Finally, the potential use of miR-9, miR-206 and miR-132 as biomarkers in SMA has been proposed. It was shown that there is differential expression of all three miRNAs in spinal cord, skeletal muscle and serum samples in SMA mice, while only miR-9 and miR-132 were differentially expressed in serum samples of SMA patients (Catapano et al., 2016).

### Amyotrophic Lateral Sclerosis (ALS)

ALS is a fatal, adult-onset, neurodegenerative disease that has an incidence of 1–2 cases in 100,000 people. ALS is characterized by the progressive loss of both UMNs and LMNs, resulting in paralysis and death 3–5 years after onset in most patients (Strong et al., 2005). About 10% of ALS cases are familial, almost always transmitted as dominant trait and frequently with high penetrance (Taylor et al., 2016).

Of the greater than 50 ALS-associated genes described to date, many are linked to RNA metabolism (Droppelmann et al., 2014). Several encode for RNA-binding proteins that have roles in miRNA biogenesis, including TAR DNA-Binding Protein 43 (TDP-43) which is a heterogenous nuclear RNP (hnRNP) that participates in RNA transcription, pre-mRNA splicing and miRNA processing (Lagier-Tourenne et al., 2010). Mutations in TDP-43 gene (*TARDBP*) account for ~4% of familial and 1.5% of sporadic ALS cases (Mackenzie et al., 2010). TDP-43 interacts with Drosha and Dicer, regulating miRNA processing at both primary and precursor levels in the nucleus and cytoplasm, respectively (Figure 2; Kawahara and Mieda-Sato, 2012).

Members of the FET family of RNA- and DNA-binding proteins, consisting of FUS/TLS, Ewing Sarcoma (EWS) Breakpoint Region 1 and TATA-Binding Protein Associated Factor 15 (TAF-15), also participate in miRNA biogenesis. FET proteins have roles in transcription, alternative splicing and in maintenance of genome stability (Svetoni et al., 2016). Of interest, all FET proteins interact with Drosha (Gregory et al., 2004). FUS/TLS binds to pri-miRNAs and helps with Drosha recruitment to chromatin for efficient miRNA processing (Figure 2; Morlando et al., 2012). EWS regulates the expression of Drosha and miRNAs, although the mechanism is unknown (Kim et al., 2014). TAF15 is required for post-transcriptional regulation of the expression of the onco-miR-17 family, which in turns controls the gene expression of cell cycle regulatory genes (Ballarino et al., 2013).

Numerous studies in ALS models, including those harboring mutations in copper/zinc superoxide dismutase (mtSOD1), and patient samples have demonstrated a disruption of miRNA expression in ALS (Williams et al., 2009; Butovsky et al., 2012; Koval et al., 2013; Parisi et al., 2013; Zhou et al., 2013; Marcuzzo et al., 2014; Toivonen et al., 2014; Dobrowolny et al., 2015). The most consistent observation from the mtSOD1 mice is the upregulation of miR-9 and miR-206 (Williams et al., 2009; Zhou et al., 2013; Toivonen et al., 2014; Dobrowolny et al., 2015). Specifically, miR-9 expression is up-regulated in mtSOD1 mouse spinal cord (Shi et al., 2004; Zhao et al., 2009; Tan et al., 2012; Zhou et al., 2013; Dobrowolny et al., 2015), while miR-206 expression is up-regulated in muscle in both mtSOD1 and SMA mouse models (Valsecchi et al., 2015). Of note, mtSOD1 disease progression is accelerated with down-regulation of miR-206 expression (Williams et al., 2009).

Dysregulation of miRNA expression has been described in spinal cord, brain, iPSC, muscle, blood and cerebrospinal fluid (CSF) of ALS patients. We obtained the first miRNA profile of human spinal cord tissue and observed a massive down-regulation of miRNAs in ALS (Campos-Melo et al., 2013). This disruption was subsequently shown to be specific to motor neurons (Emde et al., 2015).The reduction of miRNA levels has been observed to be a consequence of the inhibition of DICER pre-miRNA processing activity (Emde et al., 2015). Of note, the down-regulation of miRNAs has been observed also in other ALS-derived tissues such as in motor cortex, fibroblasts, serum /plasma and CSF (Wakabayashi et al., 2014; Freischmidt et al., 2015; Raman et al., 2015; Takahashi et al., 2015; Benigni et al., 2016). Also, a consistent up-regulation of miR-206 in muscle (Russell et al., 2013; de Andrade et al., 2016) and in serum samples of ALS patients has been reported (Toivonen et al., 2014; de Andrade et al., 2016). The latter appears to correlate with the rate of clinical deterioration (de Andrade et al., 2016). Although studies in larger cohorts are necessary, these results suggest that miR-206 could be a potential biomarker for ALS.

## Conclusion

Considering the critical function of miRNAs in neuronal differentiation, function and survival, it is easy to speculate that every neuronal cell type has to have its own miRNA profile. Motor neurons in particular are highly specialized cells that require tight regulation of gene expression for their normal function, which is in a fundamental way accomplished by MotomiRs. MotomiRs pivotal role in maintaining motor neuron homeostasis is fairly evident from the fact that alterations in their levels are linked to motor neuron malfunction and disease. Moreover, TDP-43 and FUS/TLS are well known proteins associated with ALS, but are also essential for miRNA processing, further supporting the involvement of the miRNA pathway in motor neuron impairment.

In the past few years miRNAs have emerged as the next generation of potential biomarkers and therapeutic tools for neurodegenerative diseases. The stability of miRNAs in biofluids, dysregulation in disease and their mature detection technologies make them suitable for diagnosis, disease classification and progression, and evaluation of drug effectiveness, among others. The fact that miRNAs regulate the vast majority of the transcriptome has led to the development of nanocapsules for tissue-specific delivery of miRNAs mimics and antisense oligonucleotides (anti-miRNAs) with specific targets, converting miRNAs into an attractive option for disease intervention strategies (Basak et al., 2016). Of the MotomiRs described in this review, miR-9, miR-206, miR-183 and miR-375 could potentially be explored as biomarkers and therapeutic targets for SMA and ALS, while miR-183 and miR-375 may be contributing to the specificity of SMA detection.

MiRNAs have an enormous diagnostic potential as non-invasive biomarkers and therapeutic tools of motor neuron diseases; however, we must first understand the transcriptome networks which are regulated by MotomiRs both in health and disease if we are to appreciate the complexity of this system within motor neurons. The field is still in its infancy, but the inclusion of large cohorts and specificity in the studies will certainly help to validate their use in the upcoming medicine era.

## Author Contributions

MJS contributed to the writing of the article, reviewed and approved the final submission. ZCEH contributed to the writing of the article, illustration preparation and final review. DC-M contributed to the writing of the article, illustration preparation and final review. CAD contributed to the writing of the article, illustration preparation, and final review.

## Funding

MJS research is supported by European research projects on rare diseases (E-Rare), Ontario Neurodegenerative Diseases research Initiative, the ALS Society of Canada and the Michael Halls Endowment.

## Conflict of Interest Statement

The authors declare that the research was conducted in the absence of any commercial or financial relationships that could be construed as a potential conflict of interest.
